# Record Extent and Occlusal Support in Scan-Assisted Interocclusal Registration: A Three-Dimensional In Vitro Study

**DOI:** 10.3390/bioengineering13090973

**Published:** 2026-08-25

**Authors:** Ragai Edward Matta, Paula Töpel, Fabian Lotz, Manfred Wichmann, Johannes Matthias Ries

**Affiliations:** Dental Clinic 2, Department of Prosthodontics, University Hospital Erlangen, Friedrich-Alexander-Universität Erlangen-Nürnberg (FAU), Glückstrasse 11, 91054 Erlangen, Germany; paula.toepel@uk-erlangen.de (P.T.); fabianlotz1@web.de (F.L.); claudia.ehrhardt@uk-erlangen.de (M.W.); johannes.ries@uk-erlangen.de (J.M.R.)

**Keywords:** digital dentistry, interocclusal registration, maxillomandibular relationship, intraoral scanning, virtual articulation, optical metrology, vinyl polysiloxane, three-dimensional analysis

## Abstract

Scan-assisted interocclusal registration may vary with record extent and occlusal support. Using separately fabricated high-hardness vinyl polysiloxane (VPS) records, a fixed bilateral buccal intraoral scanner (IOS) acquisition protocol, optical reference articulations, and position-level three-dimensional analysis, this in vitro study compared prepared-tooth (Hard-Prep) and complete-arch (Hard-Arch) records across three support configurations and assessed a no-record IOS workflow. Ninety VPS-based and 45 no-record IOS acquisitions were analyzed. VPS records were fabricated under a 3.0 kg load; measurements used a 1.0 kg load. Overall three-dimensional deviation (dXYZ) was averaged over five mandibular positions. Log-transformed dXYZ was analyzed by ordinary least squares with Holm-adjusted contrasts. The Hard-Arch/Hard-Prep contrast was not statistically significant under bilateral extended support. Hard-Arch showed higher dXYZ under unilateral extended support (ratio, 1.75; 95% confidence interval (CI), 1.54–1.99; *p* < 0.001) and unilateral limited support (ratio, 1.58; 95% CI, 1.39–1.80; *p* < 0.001). In the no-record workflow, unilateral extended support exceeded bilateral extended support (ratio, 1.50; 95% CI, 1.31–1.72; *p* < 0.001), whereas unilateral limited support was lower than unilateral extended support (ratio, 0.68; 95% CI, 0.59–0.77; *p* < 0.001). Prepared-tooth records yielded lower deviation in both unilateral configurations, while the no-record workflow varied with support configuration.

## 1. Introduction

Recording the maxillomandibular relationship is a critical step in digital restorative and prosthodontic workflows. Once maxillary and mandibular datasets have been articulated virtually, the generated relationship guides occlusal design and subsequent adjustment. Registration quality can therefore affect the final restoration even when the individual arch scans are dimensionally acceptable. Interocclusal registration is consequently an integral determinant of virtual articulation [1,2,3,4].

Intraoral scanner-based articulation reflects both the dimensional quality of the arch datasets and the procedure used to relate them. The acquisition strategy and subsequent digital alignment determine how the datasets are combined, while available occlusal support and record geometry define the physical information available during registration. Loading and contact conditions can further modify the generated relationship [1,2,3,4]. These factors become particularly relevant when posterior contacts are reduced or when prepared and missing teeth alter the support available during registration.

Support geometry has been investigated mainly in partially edentulous and distal-extension models. These studies showed that the span and location of missing support can affect virtual interocclusal record accuracy [5,6], while work on scanning strategies in partially edentulous arches further demonstrated the relevance of acquisition design [7]. Although these experimental settings differ from prepared-tooth configurations, they support the broader premise that the arrangement of available occlusal contacts can influence the generated maxillomandibular relationship.

The physical extent of an interposed vinyl polysiloxane (VPS) record represents a related but less studied workflow variable. Complete-arch coverage may appear advantageous because it provides a larger contact and capture area. Studies of virtual occlusal records have shown, however, that record number, dimensions, distribution, and location can affect articulation [8,9,10,11,12]. More recently, an in vitro study using three IOS systems showed that bilateral buccal registration yielded significantly lower mean spatial deviations across the complete dental arch than unilateral buccal or anterior registration [13]. Although these studies address virtual rather than physical records, they identify record geometry as a plausible determinant of the registered relationship. The performance of a complete-arch VPS record relative to a record confined to the prepared-tooth region, therefore, warrants investigation when the subsequent intraoral scanner (IOS) acquisition is held constant.

Direct no-record IOS acquisition offers a simpler alternative because it avoids an interposed material. An interposed VPS record provides a different scan-assisted approach, and its behavior may be influenced by setting characteristics, hardness, elastic response, and dimensional behavior under load [14]. Experimental studies have also demonstrated that compressive loading, optical registration conditions, and available interocclusal space can affect interocclusal registration [15,16,17]. Evaluating no-record and VPS-assisted workflows across the same support configurations may therefore clarify how registration behavior changes when the physical support geometry is altered.

Viewed as a bioengineering measurement chain, the present workflow combines controlled mechanical loading, physical record geometry, optical surface acquisition, and computational alignment. Three-dimensional analysis allows the resulting spatial discrepancy between optical reference and IOS-derived articulations to be quantified [18]. In the present study, overall three-dimensional deviation (dXYZ) served as the primary endpoint, while position-level deviations described the regional distribution of the discrepancy.

Evidence remains limited for workflows that combine standardized loading, separately fabricated high-hardness VPS records of different extents, a fixed bilateral buccal IOS acquisition, and prepared-tooth support configurations within one experimental design. The primary aim of this in vitro study was to compare overall dXYZ between prepared-tooth and complete-arch VPS records within each of three standardized model-pair configurations. A secondary, separately analyzed aim was to determine whether overall dXYZ in a no-record IOS workflow varied among the same configurations.

The null hypotheses were that (1) Hard-Prep and Hard-Arch would not differ within any configuration and (2) overall dXYZ in the no-record IOS workflow would not differ among configurations.

## 2. Materials and Methods

### 2.1. Study Design and Experimental Scope

All optical reference and workflow measurements were performed under a standardized 1.0 kg load, corresponding to approximately 9.8 N. The study included one no-record IOS workflow (Control) and two VPS workflows (Hard-Prep and Hard-Arch). VPS records were fabricated and allowed to set in the closed articulator under a standardized 3.0 kg load; this fabrication load was constant across the two VPS workflows and was not an analytical factor.

Each model-pair configuration was represented by one physical model pair, which was used for all 15 acquisitions per workflow. Each VPS acquisition used a separately fabricated record that was used once; Control comprised 15 no-record IOS acquisitions of the same physical model pair. The replication number of 15 was selected a priori after statistical consultation and was consistent with a preceding comparative in vitro study using 15 digitizations per registration strategy [13]. No formal a priori power calculation was performed. Within each model-pair series, the VPS records were fabricated before scan acquisition, after which acquisitions followed the fixed sequence Control, Hard-Prep, and Hard-Arch. All 135 planned acquisitions were completed and included; no acquisition required reacquisition, and none was excluded. Record fabrication, IOS acquisition, and GOM-based evaluation were performed by one operator.

**Figure 1:**
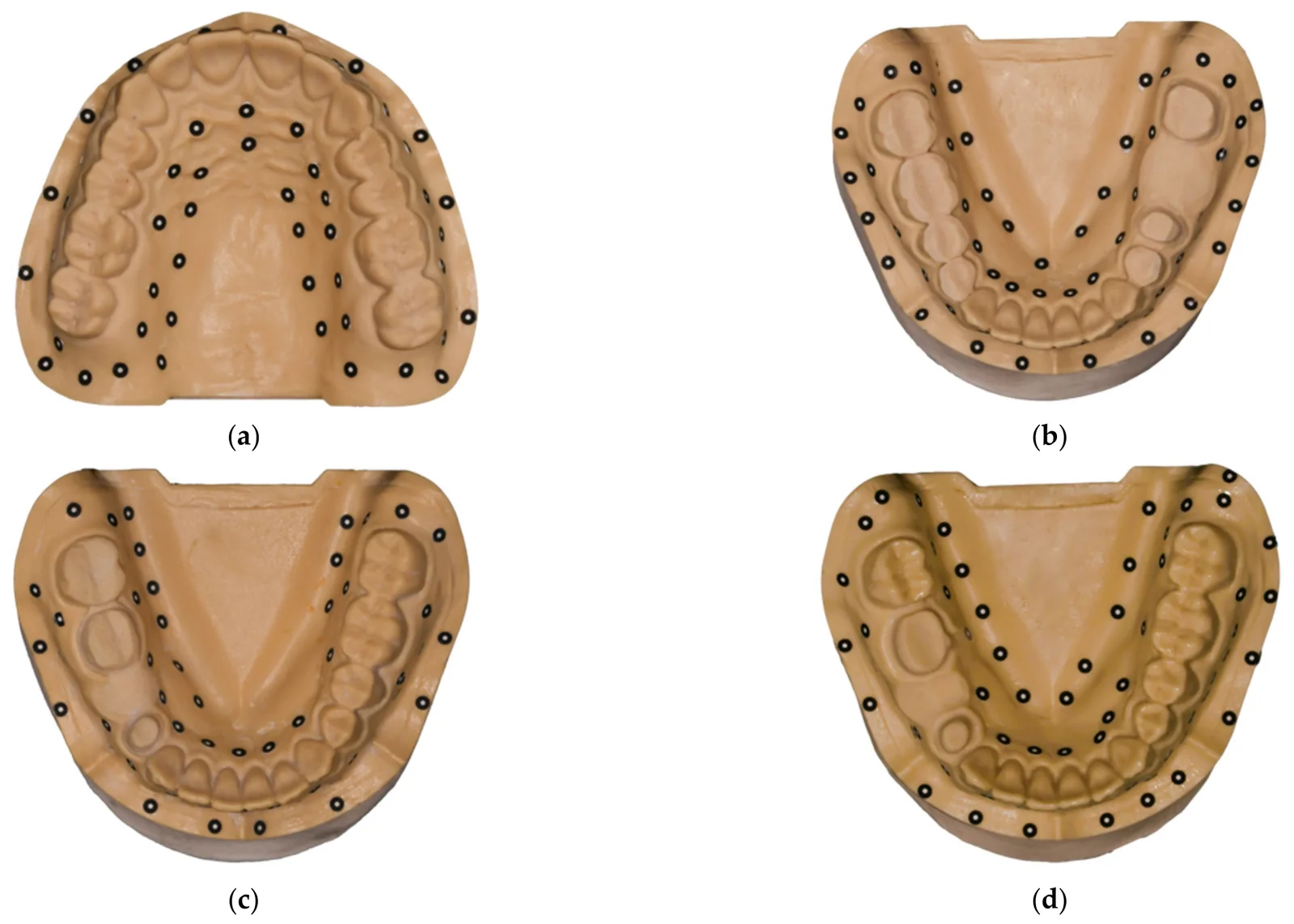

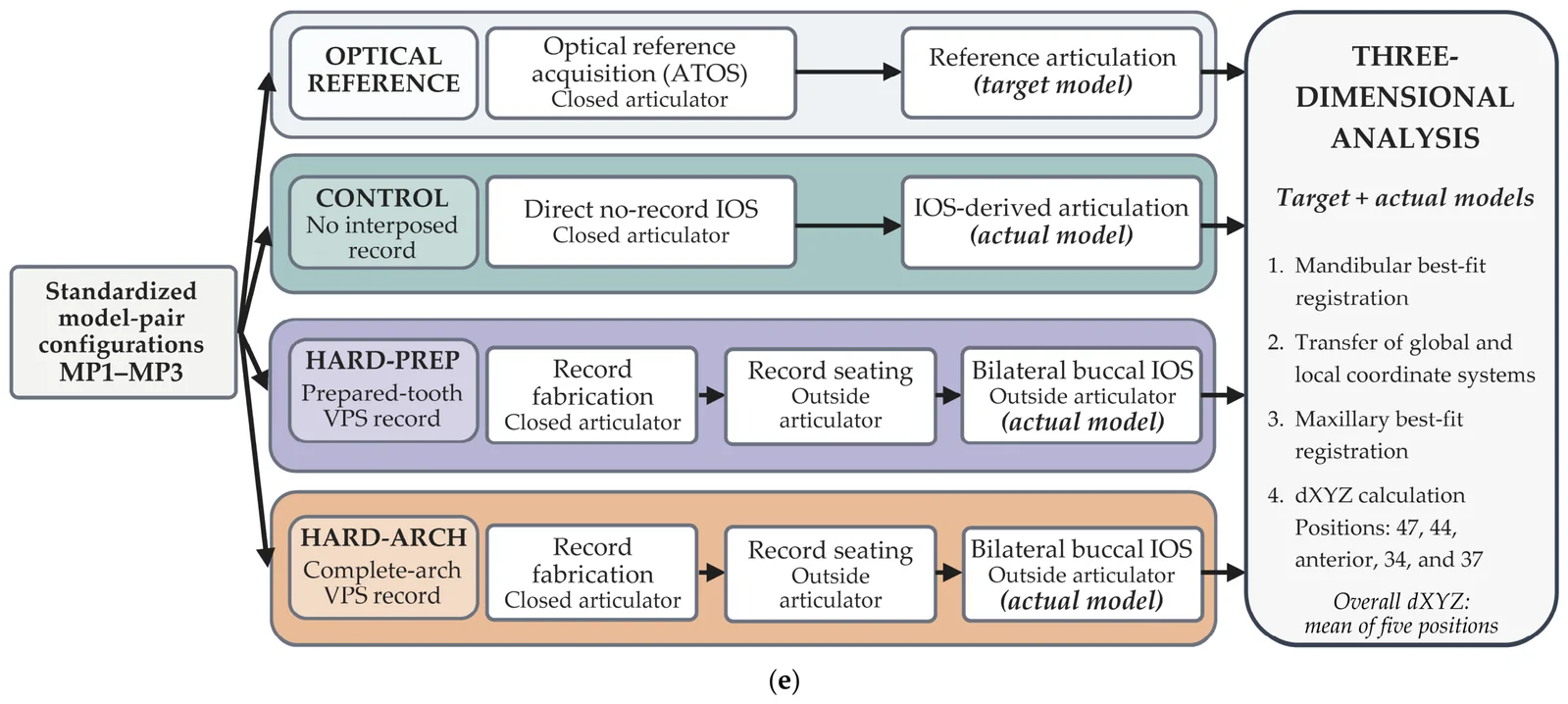
Standardized model-pair configurations and experimental workflow. (**a**) Maxillary reference model. (**b**) MP1, bilateral extended support. (**c**) MP2, unilateral extended support. (**d**) MP3, unilateral limited support. (**e**) Acquisition workflows for the optical reference, Control, Hard-Prep, and Hard-Arch conditions. Black circular markers in panels (**a**–**d**) are reference markers used for ATOS digitization. In panel (**e**), gray denotes the optical reference, teal Control, violet Hard-Prep, and orange Hard-Arch; arrows indicate the sequence of acquisition and analysis steps. IOS, intraoral scanner; MP, model-pair configuration; VPS, vinyl polysiloxane.

The primary endpoint was scan-acquisition-level overall dXYZ, calculated as the arithmetic mean of the position-level dXYZ values at five predefined mandibular measurement positions. The standardized model-pair configurations and acquisition workflows are summarized in Figure 1; workflow definitions are provided in Table 1.

### 2.2. Model-Pair Configurations

Three standardized model-pair configurations were generated and designated MP1, MP2, and MP3. The descriptors bilateral extended, unilateral extended, and unilateral limited prepared-tooth support summarize the predefined arrangement of prepared teeth, missing teeth, and remaining occlusal support surfaces within each configuration. These descriptors are distinct from VPS record extent and IOS acquisition extent. Third molars were absent in all models, and the maxillary arch was complete from Fédération Dentaire Internationale (FDI) tooth 17 to tooth 27.

MP1 included a mandibular crown preparation on tooth 37, a missing tooth 36, a crown preparation on tooth 35, a partial-crown preparation on tooth 34, and partial-crown preparations on teeth 44 to 47. All partial-crown preparations were completely reduced occlusally. MP2 included mandibular crown preparations on teeth 44 and 46, a missing tooth 45, and a partial-crown preparation on tooth 47. MP3 included mandibular crown preparations on teeth 44 and 46, with tooth 45 missing.

### 2.3. Model Preparation and Reference Digitization

The model pairs were mounted in a Protar 5 articulator (KaVo Dental GmbH, Biberach/Riss, Germany). Stable posterior occlusal contacts were established and verified using Shimstock foil before the teeth were prepared and removed according to the corresponding model-pair configuration.

One optical reference dataset was acquired for each model-pair configuration in the closed articulator under the verified 1.0 kg load using an ATOS Triple Scan industrial optical scanner (GOM GmbH, Braunschweig, Germany, a ZEISS company) and GOM Inspect Professional 2020 software. The scanner was calibrated and quality-controlled according to the manufacturer’s instructions. Reference markers were used for ATOS digitization only and were not included in matching the IOS datasets. The resulting ATOS datasets served as the metrological target models, and the subsequent registration followed the previously published three-dimensional interocclusal analysis method [18].

### 2.4. Scan-Assisted Interocclusal Registration Workflows

Control comprised direct no-record IOS acquisition with CEREC Primescan AC (Dentsply Sirona, Bensheim, Germany; software version 5.1.1) in the closed Protar 5 articulator under the verified 1.0 kg load. The optical reference articulation served as the metrological target for three-dimensional evaluation.

Hard-Prep and Hard-Arch used Futar D (Kettenbach Dental, Eschenburg, Germany; manufacturer-reported final hardness, Shore D 43) with prepared-tooth and complete-arch record extents, respectively. Material was dispensed using the same automix system and application technique. The predefined experimental factor was record extent; material volume and thickness were not quantified. For Hard-Prep, material was limited to the prepared teeth; for Hard-Arch, it extended across the complete dental arch. Records were fabricated in the closed articulator under the standardized 3.0 kg load. After complete setting, excess material was trimmed according to the manufacturer’s instructions. Before IOS acquisition, complete seating of each record was confirmed visually by gap-free adaptation and the absence of rocking. “Prep” and “Arch” refer to VPS record extent; both workflows used the same bilateral buccal IOS acquisition extent.

For both VPS workflows, the verified 1.0 kg load was applied to the seated model pair outside the articulator. No external positioning fixture was used; the maxillomandibular relationship was maintained by the fully seated VPS record engaging the available occlusal support surfaces. The articulator was intentionally omitted during IOS acquisition to evaluate the record-assisted relationship without mechanical guidance. Bilateral buccal IOS registrations were acquired outside the articulator using CEREC Primescan AC. The scanned buccal region extended from the canine to the second molar on both sides and was constant across MP1, MP2, and MP3. The maxillomandibular relationship generated by the IOS software was accepted without manual correction. All datasets were exported in Standard Tessellation Language (STL) format and imported directly into GOM Inspect Professional 2020 without mesh smoothing, mesh reduction, hole filling, or manual surface correction. Because Control and the VPS workflows differed in articulator guidance and acquisition procedure, they were analyzed as separate workflow questions; no inferential Control-versus-VPS comparison was planned.

### 2.5. Three-Dimensional Matching and Deviation Assessment

Three-dimensional deviation assessment was performed in GOM Inspect Professional 2020. The target/actual logic and surface-selection approach followed a previously described three-dimensional analysis method [18] and were adapted to the present model-pair configurations and workflows. Mandibular best-fit registration included all available dental surfaces, and all datasets permitted successful completion of the predefined matching workflow.

For each optical reference articulation, a global Cartesian coordinate system was established using a 3-2-1 alignment procedure. Five local mandibular coordinate systems were defined at tooth 47, tooth 44, the anterior region, tooth 34, and tooth 37. The same arch-relative positions were used for all configurations. X represented transverse displacement, Y sagittal displacement, and Z vertical displacement.

For each configuration and scan acquisition, the ATOS dataset served as the target model and the corresponding IOS dataset as the actual model. The mandibular target and actual meshes were prealigned and then registered by best-fit matching over the available dental surfaces. The global and local coordinate systems defined on the mandibular target were transferred to the aligned mandibular IOS model. A maxillary best-fit registration was then performed while retaining the transferred mandibular coordinate framework. The resulting displacement expressed the discrepancy between the IOS-derived and optical reference articulations.

**Figure 2:**
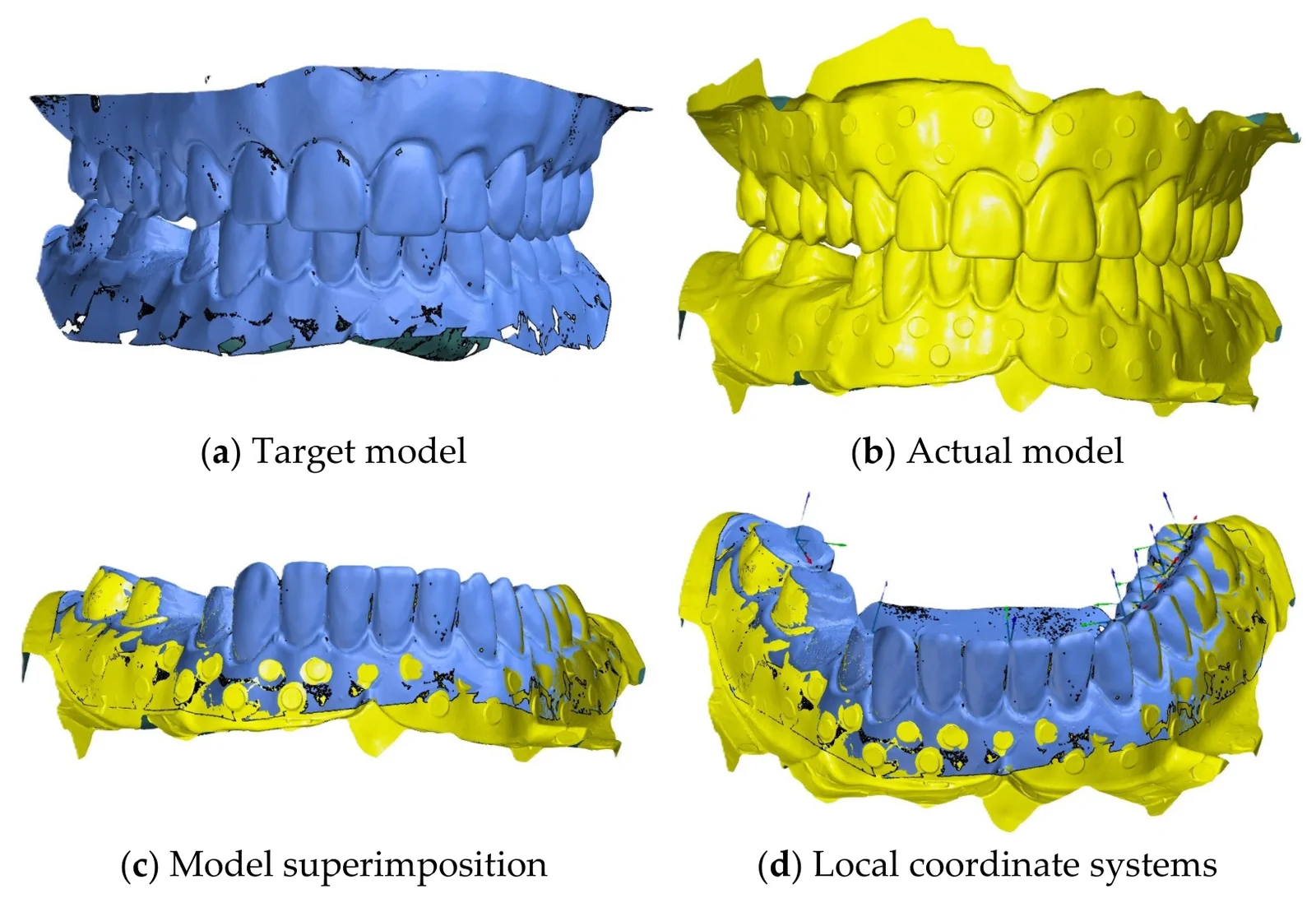
Three-dimensional matching and dXYZ determination. (**a**) Optical reference articulation used as the target model. (**b**) IOS-derived articulation used as the actual model. (**c**) Target–actual model superimposition. (**d**) Local coordinate systems used for position-level deviation assessment. Blue denotes the target model; yellow denotes the actual model. dXYZ, three-dimensional Euclidean deviation; IOS, intraoral scanner.

At each local coordinate system, translational deviations were recorded along the X-, Y-, and Z-axes. Euclidean displacement was calculated as dXYZ = √(ΔX^2^ + ΔY^2^ + ΔZ^2^). The primary endpoint, overall dXYZ, was the arithmetic mean of the five position-level values at 47, 44, the anterior region, 34, and 37. Position-level values were retained for descriptive regional analysis. The target–actual model relationship, model superimposition, and local coordinate systems used for position-level deviation assessment are illustrated in Figure 2.

### 2.6. Statistical Analysis

Analyses were performed in R version 4.6.0 (R Foundation for Statistical Computing, Vienna, Austria). Ordinary least-squares linear models were fitted with lm() to the natural logarithm of overall dXYZ. Estimated marginal means and predefined contrasts were obtained using emmeans version 2.0.3; model diagnostics and robust-covariance sensitivity checks used sandwich version 3.1.2 and lmtest version 0.9-40; figures were produced using ggplot2 version 4.0.3.

The analysis comprised 135 scan acquisitions: 90 VPS acquisitions based on separately fabricated records and 45 no-record Control acquisitions. The scan acquisition was the analysis unit. Because each configuration was represented by one physical model pair, the 15 acquisitions per workflow/configuration characterized technical workflow variability within a fixed model geometry and single-operator protocol. No missing or nonpositive overall dXYZ values were identified.

For the VPS workflows, the natural logarithm of overall dXYZ was modeled as a function of record extent, model-pair configuration, and their interaction. Predefined contrasts compared Hard-Arch with Hard-Prep within MP1, MP2, and MP3. For Control, a separate model included model-pair configuration as the fixed effect, with predefined contrasts MP2/MP1, MP3/MP1, and MP3/MP2. The Holm adjustment was applied separately within the three VPS contrasts and the three Control contrasts.

For descriptive presentation, model-based geometric means were obtained from a log-linear model including workflow, model-pair configuration, and their interaction. Estimates and ratios were back-transformed to the response scale. Reported 95% confidence intervals were unadjusted; multiplicity adjustment was applied to *p* values using the Holm method. Two-sided tests used an alpha level of 0.05. Exact *p* values are reported except for *p* < 0.001.

Model assumptions were assessed using residual-versus-fitted, normal Q-Q, scale-location, leverage, and Cook’s-distance diagnostics. Primary analyses retained all valid observations. Sensitivity analyses included a 3× interquartile range (IQR) criterion, exclusion of the global top 1% of overall dXYZ values, and HC3 heteroscedasticity-consistent robust standard errors as an internal quality-control analysis.

### 2.7. Use of Artificial Intelligence (AI)-Assisted Tools

During manuscript preparation, OpenAI ChatGPT 5.4 Pro was used to support R-script development and debugging, and OpenAI ChatGPT 5.6 Pro was used to support language editing and internal consistency checks. The statistical analyses were executed in R version 4.6.0.

## 3. Results

### 3.1. Dataset and Overall Deviation Patterns

All 135 planned scan acquisitions were included: 90 VPS acquisitions based on separately fabricated records and 45 no-record Control acquisitions. All 135 planned scan acquisitions were retained in the primary analysis. Each workflow/model-pair configuration comprised 15 acquisitions. Raw descriptive statistics are provided in Table S1.

**Figure 3:**
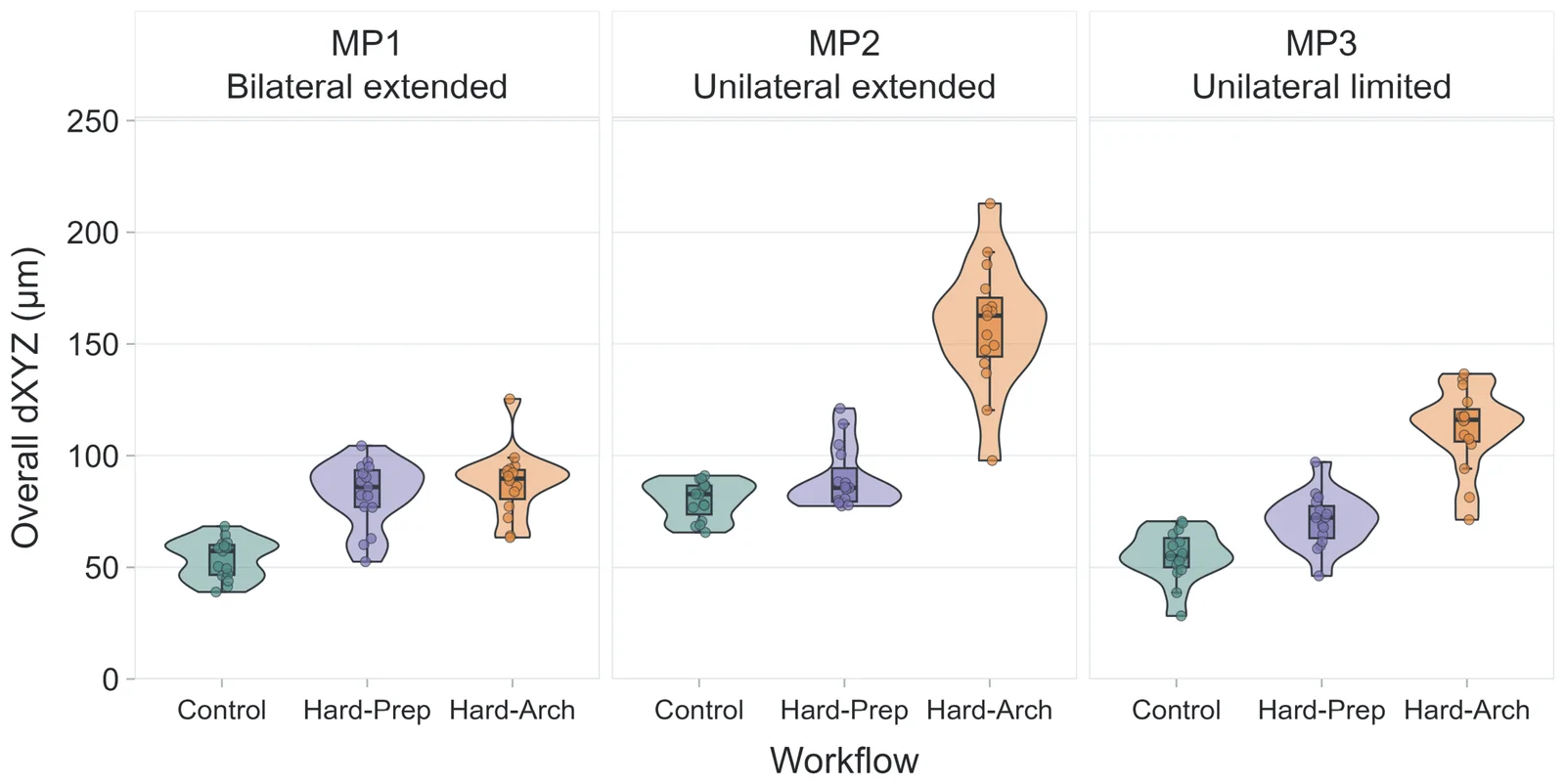
Overall dXYZ by workflow and model-pair configuration. Violin plots show the distribution of acquisition-level overall dXYZ; embedded boxplots show the median, interquartile range, and whiskers. Individual points represent the 15 scan acquisitions in each workflow/configuration. All distributions were calculated from the complete uncapped data. Model-based geometric means are reported in Table 2; predefined inferential contrasts are reported in Table 3 and Table 4. dXYZ, three-dimensional Euclidean deviation. Teal, violet, and orange denote Control, Hard-Prep, and Hard-Arch, respectively; circles represent individual scan acquisitions.

Model-based geometric means are summarized in Table 2, and the acquisition-level distributions are shown in Figure 3. In Control, the geometric mean overall dXYZ was 53.0 µm in MP1, 79.6 µm in MP2, and 53.8 µm in MP3. Hard-Prep showed values of 81.5, 88.9, and 69.8 µm in MP1, MP2, and MP3, respectively; the corresponding Hard-Arch values were 86.5, 155.5, and 110.4 µm.

**Figure 4:**
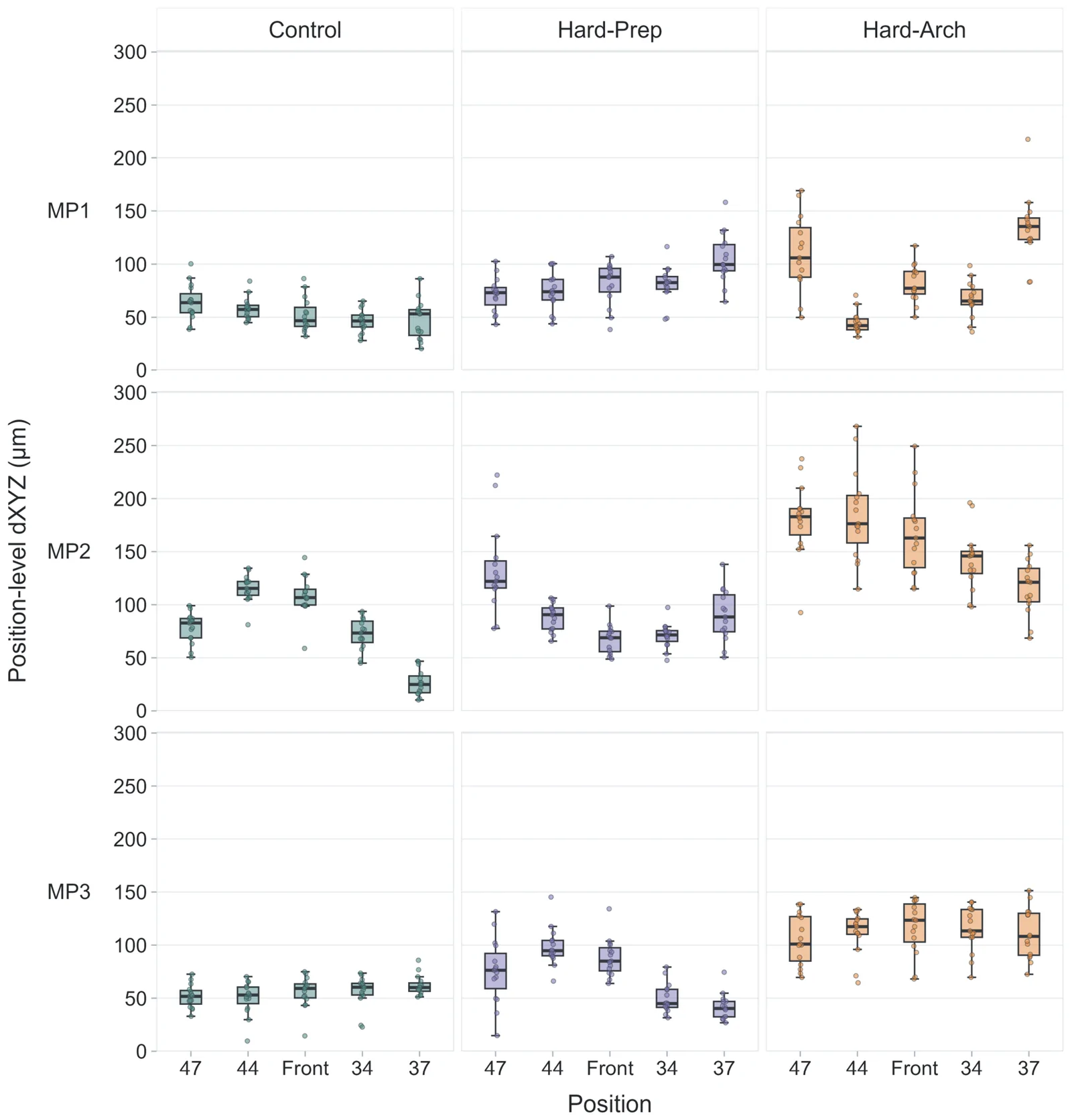
Position-level dXYZ by workflow and model-pair configuration. Boxplots show the complete position-level dXYZ distributions at mandibular positions 47, 44, the anterior region (Front), 34, and 37. Individual points represent observations from the 15 scan acquisitions in each workflow/configuration. Rows represent MP1, bilateral extended support; MP2, unilateral extended support; and MP3, unilateral limited support. Columns represent Control, Hard-Prep, and Hard-Arch. The figure provides regional description; no position-level inferential comparisons were performed. dXYZ, three-dimensional Euclidean deviation. Teal, violet, and orange denote Control, Hard-Prep, and Hard-Arch, respectively; circles represent position-level observations from individual scan acquisitions.

Position-level distributions are shown in Figure 4, and descriptive position-level geometric means and 95% confidence intervals are provided in Table S2.

**Table 3:** Predefined contrasts between the Hard-Arch and Hard-Prep workflows within each model-pair configuration.

Model-Pair Configuration	Support Configuration	Contrast	Ratio (95% CI)	Raw *p*	Holm-Adjusted *p*
MP1	bilateral extended prepared-tooth support	Hard-Arch/Hard-Prep	1.06 (0.93–1.21)	*p* = 0.357	*p* = 0.357
MP2	unilateral extended prepared-tooth support	Hard-Arch/Hard-Prep	1.75 (1.54–1.99)	*p* < 0.001	*p* < 0.001
MP3	unilateral limited prepared-tooth support	Hard-Arch/Hard-Prep	1.58 (1.39–1.80)	*p* < 0.001	*p* < 0.001

Note: Ratios represent the Hard-Arch/Hard-Prep comparison within each model-pair configuration. Ratios above 1 indicate a higher geometric mean overall dXYZ value for the Hard-Arch workflow. Holm adjustment was applied across the three predefined contrasts. Confidence intervals are unadjusted.

**Table 4:** Contrasts among model-pair configurations in the no-record IOS workflow.

Contrast	Configuration Comparison	Ratio (95% CI)	Raw *p*	Holm-Adjusted *p*
MP2/MP1	unilateral extended/bilateral extended	1.50 (1.31–1.72)	*p* < 0.001	*p* < 0.001
MP3/MP1	unilateral limited/bilateral extended	1.02 (0.89–1.16)	*p* = 0.822	*p* = 0.822
MP3/MP2	unilateral limited/unilateral extended	0.68 (0.59–0.77)	*p* < 0.001	*p* < 0.001

Note: Ratios compare model-pair configurations within Control. Holm adjustment was applied over the three predefined contrasts. Confidence intervals are unadjusted. The optical reference articulation served as the metrological target; no inferential Control-versus-VPS comparisons were performed.

### 3.2. VPS Record-Extent Contrasts

Predefined contrasts compared Hard-Arch with Hard-Prep within each model-pair configuration (Table 3). MP1 did not provide evidence of an extent-related difference (ratio, 1.06; 95% CI, 0.93–1.21; Holm-adjusted *p* = 0.357); equivalence was not assessed. Hard-Arch showed a higher geometric mean overall dXYZ than Hard-Prep in MP2 (ratio, 1.75; 95% CI, 1.54–1.99; Holm-adjusted *p* < 0.001) and MP3 (ratio, 1.58; 95% CI, 1.39–1.80; Holm-adjusted *p* < 0.001).

### 3.3. No-Record IOS Workflow

Overall dXYZ differed among the three standardized model-pair configurations in the separately analyzed no-record IOS workflow (Table 4). MP2 showed a higher geometric mean overall dXYZ than MP1 (ratio, 1.50; 95% CI, 1.31–1.72; Holm-adjusted *p* < 0.001). The MP3/MP1 contrast was not statistically significant (ratio, 1.02; 95% CI, 0.89–1.16; Holm-adjusted *p* = 0.822), whereas MP3 showed lower overall dXYZ than MP2 (ratio, 0.68; 95% CI, 0.59–0.77; Holm-adjusted *p* < 0.001).

Descriptively, position-level geometric means in MP2 were numerically higher at position 44 and in the anterior region than at position 37. No position-level inferential comparisons were performed.

## 4. Discussion

### 4.1. Principal Findings and Workflow Interpretation

Two findings define the contribution of this study. Within the tested high-hardness VPS workflows, a record limited to the prepared-tooth region yielded lower overall deviation than complete-arch coverage in both unilateral support configurations. The bilateral extended configuration showed only a small numerical difference between the two record extents. The no-record IOS workflow provided a complementary observation: its largest overall deviation occurred in the unilateral extended configuration despite the use of the same bilateral buccal scan region. Together, these observations highlight the relevance of support geometry to both record-assisted and direct optical registration.

The record-extent finding challenges the intuitive expectation that greater coverage should improve registration by providing more contact and capture area. The model-based differences between Hard-Arch and Hard-Prep were 66.6 µm in the unilateral extended configuration and 40.6 µm in the unilateral limited configuration. Complete-arch records provided a larger surface extent and potentially involved more material and record-to-tooth interfaces. Because record volume and thickness were not quantified, material volume, seating, and deformation effects remain plausible explanations rather than experimentally isolated mechanisms. The observed pattern nevertheless supports a targeted rather than size-maximized approach to physical record extent.

This interpretation depends on a clear distinction between the extent of the VPS record and the extent of IOS acquisition. Prep and Arch referred to the interposed record, whereas both VPS workflows used the same bilateral buccal scan region. Previous studies have shown that the number, dimensions, distribution, and location of virtual occlusal records can influence the generated maxillomandibular relationship [8,9,10,11,12]. Work in partially edentulous and distal-extension settings has similarly linked available support geometry to virtual interocclusal registration [5,6,7]. Those studies addressed virtual records or altered dentitions rather than a separately fabricated physical VPS record followed by a fixed bilateral IOS acquisition. The present findings add to this literature by indicating that physical record extent may remain relevant even when the subsequent scan region is held constant. The unilateral extended and unilateral limited configurations shared preparations at teeth 44 and 46 and a missing tooth 45; the unilateral extended configuration additionally included a partial-crown preparation at tooth 47. Their differing patterns should therefore be interpreted as configuration-specific responses to distinct support arrangements rather than as a simple linear effect of more versus less support.

The no-record IOS result provides a second clinically relevant perspective. Direct optical registration avoids an interposed material and can simplify the workflow, yet the geometric response varied across the three support configurations despite the use of the same bilateral buccal acquisition protocol. This result is consistent with evidence that buccal registration procedures, occlusal geometry, and digital alignment contribute to virtual articulation [8,9,19,20,21]. The optical reference articulation served as the metrological target, while Control represented direct no-record IOS registration under the same three support configurations. Its higher deviation in the unilateral extended configuration indicates that support geometry remains relevant even when the acquisition protocol itself is unchanged.

The position-level analysis helps explain the spatial character of this Control finding. In the unilateral extended configuration, descriptive values were higher around position 44 and in the anterior region than at position 37. The overall deviation, therefore, reflected a regionally concentrated pattern rather than a uniform displacement across all measurement locations. These data provide spatial context for the acquisition-level endpoint; their purpose was descriptive rather than to test a specific rotational mechanism or position-specific effect.

### 4.2. Bioengineering and Clinical Relevance

The findings are most directly applicable to tooth-supported restorative and fixed prosthodontic workflows in which crown preparations, short edentulous spans, or asymmetric posterior contacts alter the support available during registration. Two practical considerations emerge. First, complete-arch VPS coverage should not be selected solely because it provides a larger physical record. In the unilateral configurations, the prepared-tooth record yielded lower overall deviation. Because material volume and thickness were not quantified, this observation should be interpreted as a configuration-specific workflow finding rather than evidence for a particular material-volume mechanism. Second, the behavior of direct no-record IOS acquisition should be considered in relation to the available support geometry, because the same bilateral buccal scan protocol produced different geometric patterns across the three configurations. These observations support configuration-specific evaluation within each workflow rather than direct ranking of no-record and VPS-assisted approaches. A dedicated comparison would require matched acquisition conditions.

Several design features strengthen this interpretation. Each VPS acquisition was based on a separately fabricated record, the buccal scan region was kept constant across configurations, and all 135 planned acquisitions were retained in the primary analysis. The study used an optical reference articulation, predefined contrasts, separate inferential models for the VPS and Control questions, and independent raw-data verification in R. Standardized loading further controlled a procedural factor known to affect interocclusal records and optical registration [15,16,17]. Together, these measures provide a clear view of technical workflow behavior under controlled conditions.

Overall dXYZ represents an integrated workflow measure. It incorporates record fabrication and seating, IOS surface capture, software-generated articulation, STL export, mesh alignment, coordinate transfer, and GOM-based matching. This interpretation is consistent with evidence that acquisition strategy, record or scan area, and alignment repeatability contribute to virtual interocclusal registration [1,2,3,4,8,19,20,21]. The observed differences therefore reflect the complete tested workflow rather than a single material characteristic or scanner property.

The magnitude of the laboratory differences helps contextualize the relative contrasts, but their clinical meaning remains to be established. Overall dXYZ quantifies spatial disagreement between IOS-derived and optical reference articulations; it does not directly measure occlusal contact localization, adjustment time, restoration fit, patient comfort, or long-term function. Clinical studies of digital and conventional contact localization address related outcomes, but no validated threshold currently links a specific dXYZ value to a defined clinical consequence [22,23]. The present results therefore provide a quantitative basis for targeted clinical evaluation.

### 4.3. Limitations and Future Directions

The controlled scope comprised one operator, one IOS system, one VPS material, and one physical model pair per configuration. The 15 acquisitions per workflow/configuration, therefore, characterize technical workflow variability within fixed geometries. Acquisitions followed a fixed standardized sequence to maintain procedural consistency; because workflow and acquisition order were not independently varied, possible order-related effects could not be separated from workflow effects. Control and the VPS workflows also differed in articulator guidance and acquisition procedure and were therefore analyzed separately. The predefined contrasts support configuration-specific interpretation; equivalence in the bilateral extended configuration and formal comparison of contrast magnitudes across configurations were outside the planned analysis.

A calibrated ATOS system was used to acquire one fixed reference dataset per configuration, and analysis followed a published three-dimensional registration method [18]. Related dental studies have quantified the repeatability of reference alignment and triple-scan procedures [20,24]. Repeated reference acquisition and repeated GOM alignment were not part of the present protocol; therefore, the dispersion across acquisitions characterizes workflow variability relative to a fixed reference dataset and does not provide an independent estimate of reference-acquisition or alignment uncertainty. Clinical studies should assess whether the observed geometric differences translate into relevant occlusal or restorative outcomes. Further work should focus on unilateral support reduction, including direct no-record IOS registration.

## 5. Conclusions

Within the limitations of this in vitro study, scan-assisted interocclusal registration showed distinct support-dependent geometric patterns. In the unilateral extended and unilateral limited configurations, prepared-tooth high-hardness vinyl polysiloxane records yielded lower overall three-dimensional deviation than complete-arch records, while the bilateral extended configuration showed only a small numerical difference between record extents. The no-record intraoral scanner workflow also varied with support configuration and showed its greatest deviation in the unilateral extended configuration despite an unchanged bilateral buccal acquisition protocol. Overall, the findings support configuration-specific evaluation within the tested workflows; direct comparison of no-record and VPS-assisted registration requires matched acquisition conditions. Clinical studies should determine the transferability of these laboratory findings across operators, systems, model geometries, and patient-related conditions.

## Figures and Tables

**Table 1:** Workflow definitions.

Workflow	VPS Record Extent	Workflow Profile	Technical Material	Acquisition Condition
Control	not applicable	no-record IOS control	none	IOS acquisition in the closed articulator under the predefined 1.0 kg measurement load; optical reference articulation served as target
Hard-Prep	prepared teeth	high-final-hardness VPS profile	Futar D	Bilateral buccal IOS registration with the seated VPS record under the predefined 1.0 kg measurement load outside the articulator
Hard-Arch	complete arch	high-final-hardness VPS profile	Futar D	Bilateral buccal IOS registration with the seated VPS record under the predefined 1.0 kg measurement load outside the articulator

Note: Control denotes the no-record IOS workflow. The optical reference articulation served as the metrological target. Prep and Arch refer to the extent of the interposed VPS record. Product names are provided for technical reproducibility.

**Table 2:** Model-based overall dXYZ by workflow and model-pair configuration.

Workflow	VPS Record Extent	Model-Pair Configuration	Support Configuration	*n*	Geometric Mean (95% CI), µm
Control	not applicable	MP1	bilateral extended prepared-tooth support	15	53.0 (48.4–58.1)
Hard-Prep	prepared teeth	MP1	bilateral extended prepared-tooth support	15	81.5 (74.3–89.3)
Hard-Arch	complete arch	MP1	bilateral extended prepared-tooth support	15	86.5 (78.9–94.8)
Control	not applicable	MP2	unilateral extended prepared-tooth support	15	79.6 (72.7–87.3)
Hard-Prep	prepared teeth	MP2	unilateral extended prepared-tooth support	15	88.9 (81.1–97.4)
Hard-Arch	complete arch	MP2	unilateral extended prepared-tooth support	15	155.5 (141.9–170.4)
Control	not applicable	MP3	unilateral limited prepared-tooth support	15	53.8 (49.1–59.0)
Hard-Prep	prepared teeth	MP3	unilateral limited prepared-tooth support	15	69.8 (63.7–76.5)
Hard-Arch	complete arch	MP3	unilateral limited prepared-tooth support	15	110.4 (100.7–120.9)

Note: *n* denotes the number of scan acquisitions. Values are back-transformed geometric means with unadjusted 95% confidence intervals. CI, confidence interval.

## Data Availability

The raw data supporting the conclusions of this article will be made available by the authors on request.

## Supplementary Materials

The following supporting information can be downloaded at: https://www.mdpi.com/article/10.3390/bioengineering13090973/s1. Table S1, Overall dXYZ descriptive statistics; Table S2, Position-level descriptive geometric means and 95% confidence intervals; Table S3, Model quality-control summary; Table S4, Sensitivity analysis summary.

## Abbreviations

The following abbreviations are used in this manuscript:

AI	Artificial intelligence
CI	Confidence interval
dXYZ	Three-dimensional Euclidean deviation
FDI	Fédération Dentaire Internationale tooth-numbering system
HC3	Heteroscedasticity-consistent covariance matrix estimator, type 3
IOS	Intraoral scanner
IQR	Interquartile range
MP	Model-pair configuration
STL	Standard Tessellation Language
VPS	Vinyl polysiloxane
