# Supplementary material for: Record Extent and Occlusal Support in Scan-Assisted Interocclusal Registration: A Three-Dimensional In Vitro Study
Source: Bioengineering (Basel). 2026 Aug 25;13(9):973. doi: 10.3390/bioengineering13090973 (PMC13603494; doi:10.3390/bioengineering13090973)
Supplement: Supplementary file 1 [file bioengineering-13-00973-s001.zip › bioengineering-4511814-supplementary.pdf]

Supplementary Materials

Record Extent and Occlusal Support in Scan-Assisted Inter-occlusal Registration: A Three-Dimensional In Vitro Study

Ragai Edward Matta, Paula Töpel, Fabian Lotz, Manfred Wichmann, and Johannes Matthias Ries

Table S1. Overall dXYZ descriptive statistics for Control, Hard-Prep, and Hard-Arch.

Workflow	Configuration	n	Arithmetic mean, µm	SD, µm	Geometric mean, µm	Median, µm	IQR, µm	Minimum, µm	Maximum, µm
**Control**	MP1	15	53.7	9.0	53.0	57.3	13.4	38.9	68.4
**Hard-Prep**	MP1	15	82.8	14.8	81.5	86.0	16.5	52.5	104.4
**Hard-Arch**	MP1	15	87.7	15.2	86.5	89.7	13.0	63.3	125.4
**Control**	MP2	15	80.1	8.5	79.6	82.8	12.9	65.6	91.1
**Hard-Prep**	MP2	15	89.8	13.9	88.9	85.6	14.9	77.4	121.2
**Hard-Arch**	MP2	15	158.0	28.4	155.5	162.7	26.4	97.8	212.9
**Control**	MP3	15	55.1	11.5	53.8	55.1	13.1	28.3	70.6
**Hard-Prep**	MP3	15	70.8	12.2	69.8	72.3	14.4	46.2	97.1
**Hard-Arch**	MP3	15	111.9	18.4	110.4	116.0	14.5	71.3	136.7

Note: Statistics are based on the complete acquisition-level dataset. SD, standard deviation; IQR, interquartile range.

Table S2. Position-level descriptive geometric means and distribution summaries.

Workflow	Configuration	Position	n	Geometric mean (95% CI), μm	Median, μm	IQR, μm	Minimum, μm	Maximum, μm
**Control**	MP1	47	15	61.8 (53.4-71.5)	63.8	17.8	38.8	100.3
**Control**	MP1	44	15	57.5 (52.4-63.1)	57.4	10.7	45.0	84.0
**Control**	MP1	Front	15	50.3 (42.9-59.0)	46.8	17.9	32.1	86.4
**Control**	MP1	34	15	45.5 (39.9-51.9)	46.6	11.2	28.1	65.2
**Control**	MP1	37	15	44.1 (35.2-55.3)	53.2	24.2	20.5	86.2
**Hard-Prep**	MP1	47	15	69.5 (61.0-79.3)	73.1	16.4	43.3	102.5
**Hard-Prep**	MP1	44	15	72.3 (62.7-83.5)	74.1	19.3	43.9	100.4
**Hard-Prep**	MP1	Front	15	78.9 (67.2-92.8)	87.9	22.2	38.5	107.1
**Hard-Prep**	MP1	34	15	79.3 (69.9-90.0)	82.6	11.8	48.3	116.5
**Hard-Prep**	MP1	37	15	103.0 (90.9-116.6)	99.6	24.6	64.6	158.1
**Hard-Arch**	MP1	47	15	104.5 (86.1-126.9)	105.9	46.6	49.9	169.1
**Hard-Arch**	MP1	44	15	44.0 (39.2-49.4)	42.2	10.2	31.6	70.8
**Hard-Arch**	MP1	Front	15	80.5 (71.2-91.1)	77.4	21.2	50.2	117.2
**Hard-Arch**	MP1	34	15	64.8 (55.8-75.3)	65.3	14.2	36.3	98.6
**Hard-Arch**	MP1	37	15	130.8 (115.0-148.8)	135.4	20.3	83.1	217.4
**Control**	MP2	47	15	76.7 (68.7-85.6)	82.8	18.3	50.5	99.1
**Control**	MP2	44	15	114.4 (106.9-122.4)	115.4	13.0	81.1	134.4
**Control**	MP2	Front	15	106.6 (95.6-119.0)	106.7	14.8	58.9	144.4
**Control**	MP2	34	15	70.8 (62.6-80.1)	73.3	20.1	45.0	93.6
**Control**	MP2	37	15	23.6 (18.1-30.8)	24.9	15.8	10.2	46.8
**Hard-Prep**	MP2	47	15	127.1 (108.0-149.5)	122.0	25.5	77.7	222.2
**Hard-Prep**	MP2	44	15	87.3 (80.3-94.9)	90.6	19.8	65.7	106.3

**Hard-Prep**	MP2	Front	15	66.3 (59.3-74.1)	68.9	19.3	48.8	98.7
**Hard-Prep**	MP2	34	15	69.9 (63.6-76.8)	71.5	10.2	47.6	97.5
**Hard-Prep**	MP2	37	15	86.9 (74.3-101.6)	88.5	34.8	50.4	138.0
**Hard-Arch**	MP2	47	15	176.7 (156.4-199.5)	182.9	24.7	92.6	237.4
**Hard-Arch**	MP2	44	15	180.4 (158.8-205.0)	176.3	44.7	114.8	268.1
**Hard-Arch**	MP2	Front	15	162.9 (143.2-185.3)	162.8	46.8	115.0	249.3
**Hard-Arch**	MP2	34	15	139.2 (124.8-155.3)	145.9	20.9	98.1	196.0
**Hard-Arch**	MP2	37	15	113.4 (99.4-129.3)	121.2	31.5	68.6	155.9
**Control**	MP3	47	15	50.5 (44.8-56.9)	51.7	12.8	33.0	72.6
**Control**	MP3	44	15	46.4 (35.4-60.8)	53.0	15.6	9.6	70.3
**Control**	MP3	Front	15	52.7 (42.3-65.6)	59.2	13.2	14.5	74.9
**Control**	MP3	34	15	53.6 (44.1-65.0)	60.3	11.0	22.8	73.6
**Control**	MP3	37	15	61.4 (56.8-66.5)	60.0	7.8	51.2	85.7
**Hard-Prep**	MP3	47	15	67.8 (50.2-91.5)	76.3	33.2	14.7	131.5
**Hard-Prep**	MP3	44	15	96.8 (87.7-106.7)	94.7	14.5	66.1	145.3
**Hard-Prep**	MP3	Front	15	86.7 (77.9-96.4)	84.9	21.8	63.9	134.2
**Hard-Prep**	MP3	34	15	48.4 (41.8-56.1)	45.0	17.0	31.6	79.3
**Hard-Prep**	MP3	37	15	40.5 (35.0-46.9)	40.3	14.5	26.9	74.5
**Hard-Arch**	MP3	47	15	102.3 (89.9-116.5)	101.0	41.9	69.5	138.6
**Hard-Arch**	MP3	44	15	109.8 (97.5-123.7)	117.4	14.5	64.5	133.4
**Hard-Arch**	MP3	Front	15	113.9 (99.4-130.3)	123.4	35.8	68.1	144.8
**Hard-Arch**	MP3	34	15	113.1 (101.0-126.6)	113.4	26.2	69.6	140.5
**Hard-Arch**	MP3	37	15	108.4 (96.1-122.4)	108.2	39.5	72.5	151.3

Note: Position-level summaries are descriptive. No position-level inferential comparisons were performed. CI, confidence interval; IQR, interquartile range.

Table S3. Model quality-control summary.

Model	n	Parameters	Residual df	Cook flags	Leverage flags	Studentized flags	Any influential	Formula
**VPS primary model**	90	6	84	5	0	0	5	ln(overall dXYZ) ~ record extent × configuration
**Control model**	45	3	42	1	0	1	1	ln(overall dXYZ) ~ configuration

Note: Influence flags were used for quality control; all valid observations remained in the primary analyses.

Table S4. Sensitivity analysis summary.

Scenario	Analysis	Contrast	Configuration	n	Variance estimator	Ratio (95% CI)	Holm-adjusted p
**Primary: all valid observations**	VPS workflows	Hard-Arch / Hard-Prep	MP1	90	Conventional OLS	1.06 (0.93-1.21)	p = 0.357
**Primary: all valid observations**	VPS workflows	Hard-Arch / Hard-Prep	MP2	90	Conventional OLS	1.75 (1.54-1.99)	p < 0.001
**Primary: all valid observations**	VPS workflows	Hard-Arch / Hard-Prep	MP3	90	Conventional OLS	1.58 (1.39-1.80)	p < 0.001
**Primary: all valid observations**	Control	MP2 / MP1	—	45	Conventional OLS	1.50 (1.31-1.72)	p < 0.001
**Primary: all valid observations**	Control	MP3 / MP1	—	45	Conventional OLS	1.02 (0.89-1.16)	p = 0.822
**Primary: all valid observations**	Control	MP3 / MP2	—	45	Conventional OLS	0.68 (0.59-0.77)	p < 0.001
**3-IQR sensitivity**	VPS workflows	Hard-Arch / Hard-Prep	MP1	90	Conventional OLS	1.06 (0.93-1.21)	p = 0.357
**3-IQR sensitivity**	VPS workflows	Hard-Arch / Hard-Prep	MP2	90	Conventional OLS	1.75 (1.54-1.99)	p < 0.001
**3-IQR sensitivity**	VPS workflows	Hard-Arch / Hard-Prep	MP3	90	Conventional OLS	1.58 (1.39-1.80)	p < 0.001
**3-IQR sensitivity**	Control	MP2 / MP1	—	45	Conventional OLS	1.50 (1.31-1.72)	p < 0.001
**3-IQR sensitivity**	Control	MP3 / MP1	—	45	Conventional OLS	1.02 (0.89-1.16)	p = 0.822
**3-IQR sensitivity**	Control	MP3 / MP2	—	45	Conventional OLS	0.68 (0.59-0.77)	p < 0.001
**Global top-1% sensitivity**	VPS workflows	Hard-Arch / Hard-Prep	MP1	88	Conventional OLS	1.06 (0.94-1.21)	p = 0.348
**Global top-1% sensitivity**	VPS workflows	Hard-Arch / Hard-Prep	MP2	88	Conventional OLS	1.68 (1.47-1.92)	p < 0.001
**Global top-1% sensitivity**	VPS workflows	Hard-Arch / Hard-Prep	MP3	88	Conventional OLS	1.58 (1.39-1.79)	p < 0.001

**Global top-1% sensitivity**	Control	MP2 / MP1	—	45	Conventional OLS	1.50 (1.31-1.72)	$p < 0.001$
**Global top-1% sensitivity**	Control	MP3 / MP1	—	45	Conventional OLS	1.02 (0.89–1.16)	$P = 0.822$
**Global top-1% sensitivity**	Control	MP3 / MP2	—	45	Conventional OLS	0.68 (0.59–0.77)	$P < 0.001$

Note: Primary analyses retained all valid observations. Sensitivity analyses used the 3-IQR and global top-1% criteria and HC3 robust standard errors. HC3 results were used for quality control and did not replace the primary ordinary least-squares analyses.
